# PRDX4 mitigates diabetic retinopathy by inhibiting reactive gliosis, apoptosis, ER stress, oxidative stress, and mitochondrial dysfunction in Müller cells

**DOI:** 10.1016/j.jbc.2024.108111

**Published:** 2024-12-18

**Authors:** Yue Huang, Yuting Zhang, Yuan Liu, Yinan Jin, Hongwei Yang

**Affiliations:** Department of Ophthalmology, Shengjing Hospital of China Medical University, Shenyang, Liaoning, China

**Keywords:** PRDX4, diabetic retinopathy, Müller cell, reactive gliosis, endoplasmic reticulum stress, mitochondrial dysfunction

## Abstract

Diabetic retinopathy (DR) is a neurovascular complication of diabetes. As a crucial player in the retinal physiology, Müller cells are affected in DR, impairments of Müller cell function lead to retinal malfunctions. Therefore, searching for approaches to mitigate diabetes-induced injury in Müller cells is imperative for delaying DR. Peroxiredoxin 4 (PRDX4), an important endoplasmic reticulum (ER)–resident antioxidant, was explored in this study for its potential protective role against DR. Streptozotocin-induced mouse model of diabetes and high glucose (HG)–induced Müller cells were utilized to assess the impact of PRDX4. Compared with WT mice, PRDX4 knockout exacerbated retinal neurodegeneration, reactive gliosis, cell apoptosis, ER stress, oxidative stress, and mitochondrial dysfunction in diabetic retinas. Knockdown of PRDX4 aggravated HG-induced reactive gliosis, apoptosis, ER stress, oxidative stress, and mitochondrial dysfunction in Müller cells. Conversely, PRDX4 overexpression in Müller cells protected against HG-induced cell damage. Mechanistically, PRDX4 promoted the degradation of dipeptidyl peptidase-4, which is associated with DR in type 1 diabetics, thereby alleviating HG-stimulated Müller cell abnormalities. Our study indicated that PRDX4 is a crucial protective regulator in DR progression *via* destabilization of dipeptidyl peptidase-4 protein and suggested that enhancement of PRDX4 level may represent a promising approach for treating DR.

Diabetic retinopathy (DR) represents a prevalent microvascular complication associated with diabetes, emerging as the primary contributor to visual impairment among both working-age individuals and elderly individuals worldwide (1, 2). Statistics suggest that about 75% of patients with type 1 diabetes and 50% of patients with type 2 diabetes will manifest symptoms of DR (3). The pathogenesis of DR is multifaceted, including proinflammatory changes, microglia activation, oxidative stress, mitochondrial dysfunction, and cell apoptosis (4, 5, 6, 7, 8). These processes culminate in impaired function and structure of multiple retinal cell types, which lead to vasoregression, retinal neurodegeneration, neovascularization, thereby causing significant dysfunction of blood–retinal barrier in a hyperglycemic environment (9). As the main neuroglia spanning the entire neuroretina, Müller cells have a critical role in the maintenance of retina homeostasis by supplying nutrients and ensuring structural stability within the retina (10). Under hyperglycemic condition, impairment of Müller cells induces injury to the neuroretina.

Peroxiredoxins (PRDXs) constitute a group of proteins serving as peroxidases, featuring an active site cysteine susceptible to oxidation by hydrogen peroxide (11). Within the PRDX family members, PRDX4 is unique in possessing N-terminal hydrophobic amino acid sequences that are critical for secretion or retention by endoplasmic reticulum (ER) lumen (12). After elimination of hydrogen peroxide, PRDX4 facilitates oxidative protein folding by oxidizing protein disulfide isomerases, thereby mitigating ER oxidative stress (13, 14). Recent researches have linked PRDX4 to diverse diseases, including nonalcoholic steatohepatitis, atherosclerosis, colitis, ovarian ageing, among others (15, 16, 17, 18, 19). Noteworthy findings demonstrated that PRDX4 overexpression also improves insulin synthesis and secretion in pancreatic β-cells (20). PRDX4 overexpression in mice relieves streptozotocin (STZ)-induced diabetes by repressing oxidative stress and inflammatory responses (21). PRDX4 ameliorates diabetic cardiomyopathy by limiting oxidative stress and apoptosis of cardiomyocytes (22). Considering the protective role of PRDX4 in diabetes and its complications, we reasoned that PRDX4 may be involved in DR development and set out to investigate the exact role of PRDX4 in DR.

Dipeptidyl peptidase-4 (DPP4) is an enzyme classified as a serine exopeptidase, found either as a membrane-anchored cell surface protein or in a soluble state in the peripheral circulation. Its enzymatic function involves cleaving X-proline dipeptides from the N terminus of polypeptides (23). DPP4 has been proven to participate in multiple diseases, with potential mechanisms including oxidative stress, ER stress, cell apoptosis, mitochondrial dysfunction, and immune imbalance (24, 25, 26, 27). Elevated DPP4 activity has been detected in diabetic individuals (28, 29), and its circulating levels have been linked to DR in type 1 diabetes (30). Studies suggest that DPP4 inhibitors may offer therapeutic benefits for DR (31, 32). The BioGRID database has documented an interaction between PRDX4 and DPP4. It has aroused our interest regarding the potential regulatory role of PRDX4 on DPP4 in DR progression.

In the current study, C57BL/6J (WT) and PRDX4-KO mice were administered STZ injection to explore the involvement of PRDX4 in DR. Through treatment of Müller cells with high glucose (HG), the impact of PRDX4 knockdown/overexpression on reactive gliosis, cell apoptosis, ER stress, oxidative stress, and mitochondrial dysfunction was elucidated. We also give distinct insights that PRDX4 regulated the progression of DR through destabilization of DPP4 protein.

## Results

### PRDX4 deficiency increased retinal neurodegeneration and glial cell activation in STZ-induced diabetic mice

**Figure 1:**
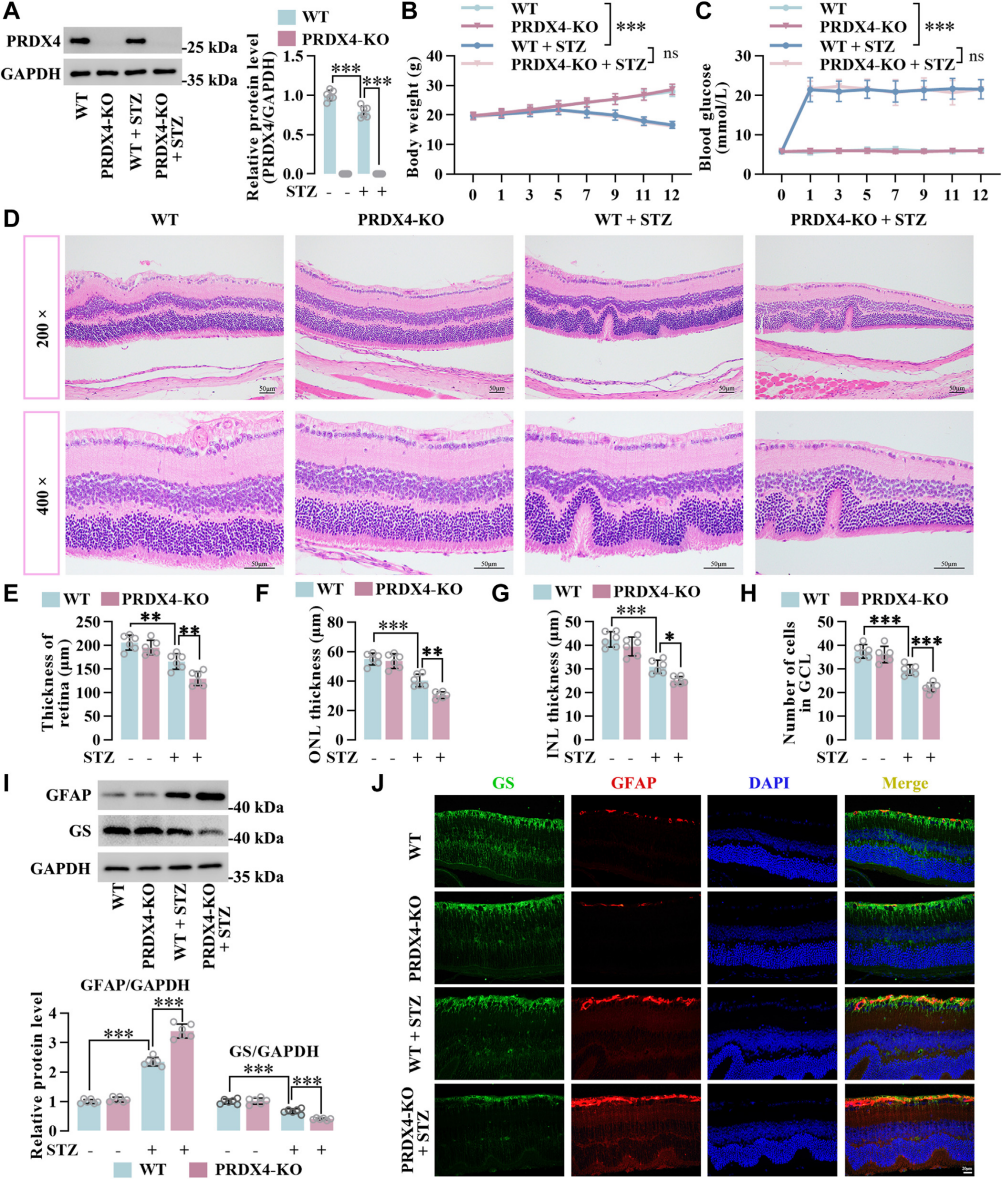
**PRDX4 deficiency increased retinal neurodegeneration and glial cell activation in STZ-induced diabetic mice**. *A*, the expression of PRDX4 in the retinas of diabetic mice (blood glucose levels >16.7 mmol/l) was determined by Western blot analysis. *B* and *C*, body weight and blood glucose level in nondiabetic and diabetic WT and PRDX4-KO mice. *D*, *H*&*E* staining showed retinal morphology and integrity in nondiabetic and diabetic WT and PRDX4-KO mice. Scale bar represents 50 μm. *E*–*G*, total retinal thickness and thickness of each retinal layer were measured. *H*, number of cells in ganglionic cell layer (GCL). *I*, protein level of GFAP and GS in retinas of nondiabetic and diabetic WT and PRDX4-KO mice determined by Western blot analysis. *J*, reactive gliosis of Müller cells in retinas was identified by GFAP and GS immunofluorescence staining. Scale bar represents 20 μm. All data are shown as mean ± SD. ∗*p* < 0.05, ∗∗*p* < 0.01, ∗∗∗*p* < 0.001 by one-way ANOVA with Tukey multiple comparison test or two-way ANOVA. GFAP, glial fibrillary acidic protein; GS, glutamine synthetase; INL, inner nuclear layer; ONL, outer nuclear layer; PRDX4, peroxiredoxin 4; STZ, streptozotocin.

To investigate the potential role of PRDX4 in DR, we utilized a loss-of-function approach by genetically ablating PRDX4 in mice under diabetic and nondiabetic conditions. Western blot analysis indicated a reduction in PRDX4 expression in the retinas of diabetic mice compared with nondiabetic mice (Fig. 1*A*). After STZ induction, diabetic mice from WT and PRDX4-KO groups exhibited lower body weights compared with their respective controls (Fig. 1*B*). Blood glucose levels were significantly elevated in diabetic mice compared with control groups (Fig. 1*C*). There was no significant difference in body weight/blood glucose between diabetic WT and diabetic PRDX4-KO mice (Fig. 1, *B* and *C*). Histochemical staining identified that STZ-induced hyperglycemia decreased the overall retinal thickness, outer nuclear layer thickness, and inner nuclear layer thickness, accompanied by a reduced number of cells in the ganglion cell layer. PRDX4 deficiency exacerbated neurodegeneration in the retina under hyperglycemia (Fig. 1, *D*–*H*). These data suggested that PRDX6 deficiency aggravated STZ-induced retinal neurodegeneration in mice.

To explore whether glial cell activation (termed reactive gliosis) in DR is mediated by PRDX4, Western blot analysis for the gliotic marker glial fibrillary acidic protein (GFAP) in retinas of mice was performed. The level of GFAP was increased in STZ-induced mice, with a further increase found in diabetic PRDX4-KO mice than in diabetic mice. We found a reduction in the level of glutamine synthetase (GS), a marker for Müller cells, in the retinas of diabetic mice. GS expression was lower in the retinas of PRDX4-KO mice compared with WT mice under diabetic conditions (Fig. 1*I*). To validate the increase of GFAP expression occurring in Müller cells, double immunofluorescence staining for GFAP and GS was conducted on retinal sections. A rise in GFAP immunoreactivity in GS-positive cells was observed following STZ induction. The immunoreactivity level was higher in activated Müller cells (GS and GFAP-positive cells) in diabetic PRDX4-KO mice compared with diabetic mice (Fig. 1*J*). These data demonstrated that PRDX4 deficiency exacerbated the diabetes-induced reactive gliosis in Müller cells.

### PRDX4 deficiency facilitated cell apoptosis and ER stress in the retinas of STZ-induced diabetic mice

**Figure 2:**
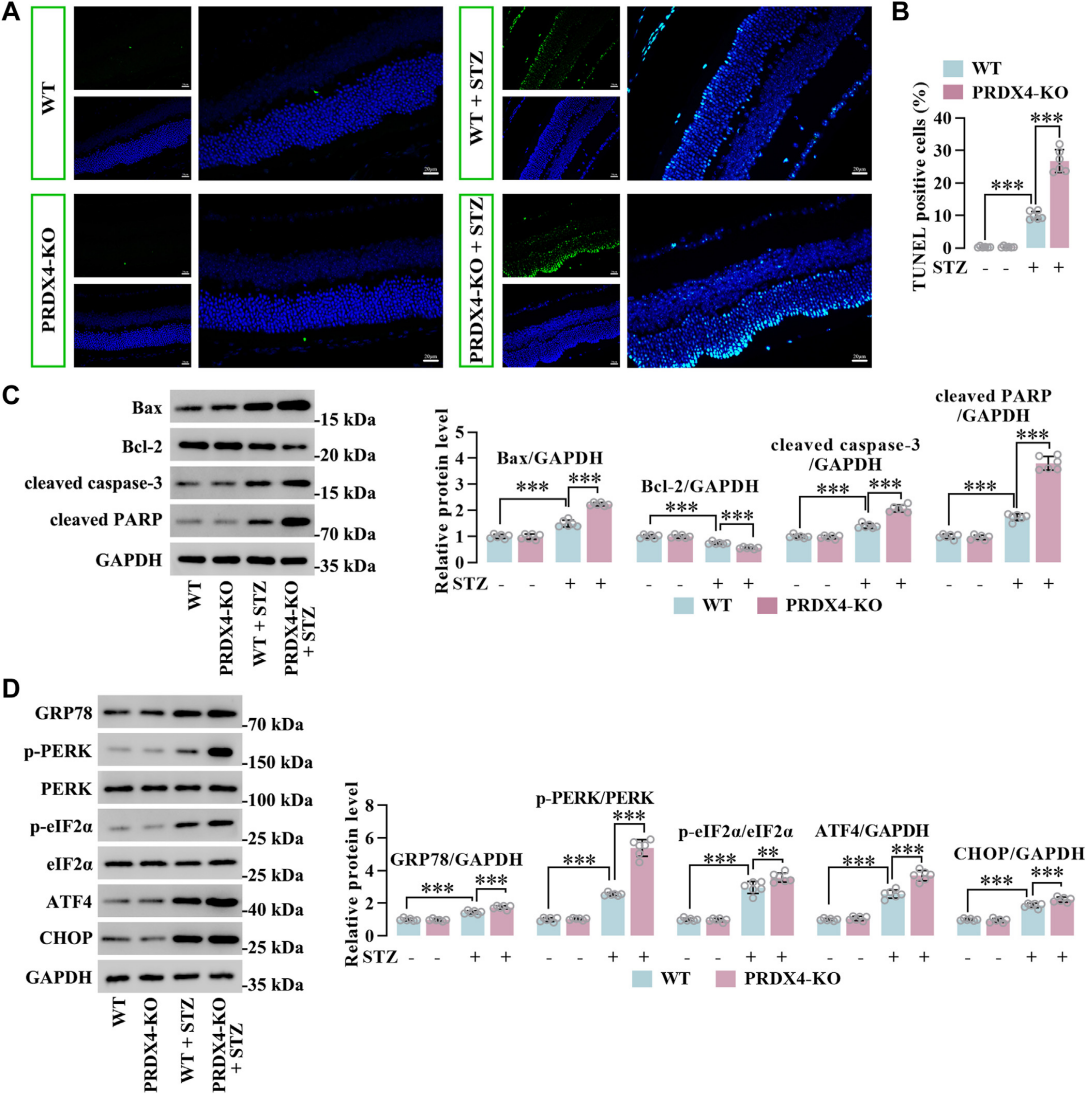
**PRDX4 deficiency facilitated cell apoptosis and ER stress in the retinas of STZ-induced diabetic mice**. *A* and *B*, TUNEL staining was conducted at 12 weeks after diabetes induction. Scale bar represents 20 μm. *C*, Western blot analysis of Bax, cleaved caspase-3, cleaved PARP, and Bcl-2 in retinas of nondiabetic and diabetic WT and PRDX4-KO mice. *D*, protein levels of GRP78, ATF4, CHOP, PERK, and eIF2α and phosphorylation levels of PERK and eIF2α in retinas of nondiabetic and diabetic WT and PRDX4-KO mice. All data are shown as mean ± SD. ∗∗∗*p* < 0.001 by one-way ANOVA with Tukey multiple comparison test. ER, endoplasmic retciclum; PARP, poly(ADP-ribose) polymerase; PRDX4, peroxiredoxin 4; STZ, streptozotocin.

TUNEL assay revealed that WT and PRDX4-KO mice displayed a minimal amount of apoptotic cells in the retinas in normal physiological conditions. However, mice showed an increase in apoptotic cells under diabetic conditions, particularly in the PRDX4-KO mice where the number of apoptotic cells exceeded that observed in WT mice (Fig. 2, *A* and *B*). In retinas of diabetic mice, levels of proapoptotic Bax, cleaved caspase-3, and cleaved poly(ADP-ribose) polymerase (PARP) were increased, concomitant with reduced antiapoptotic Bcl-2 expression compared with those of retinas from WT mice. Interestingly, retinas of diabetic PRDX4-KO exhibited further elevation in Bax, cleaved caspase-3, and cleaved PARP level, further decrease in Bcl-2 expression compared with those of diabetic mice (Fig. 2*C*). These data suggested that PRDX4 deficiency facilitated cell apoptosis in the retinas of diabetic mice.

To understand the molecular pathology of PRDX4 deficiency–induced retinal injury in diabetes, we determined the levels of ER stress–associated markers in mouse retinas. The expression of GRP78, ATF4, and CHOP, as well as the phosphorylation levels of PERK (protein kinase R-like endoplasmic reticulum) and eIF2α (eukaryotic initiation factor-2α), were found to be elevated in the retinas of diabetic mice in comparison to WT mice. However, the expression and phosphorylation levels of these protein markers were further enhanced in retinas of diabetic PRDX4-KO mice compared with diabetic mice (Fig. 2*D*). These data indicated that PRDX4 deficiency induced ER stress in the retinas of diabetic mice.

### PRDX4 deficiency contributed to mitochondria dysfunction and oxidative stress in the retinas of STZ-induced diabetic mice

**Figure 3:**
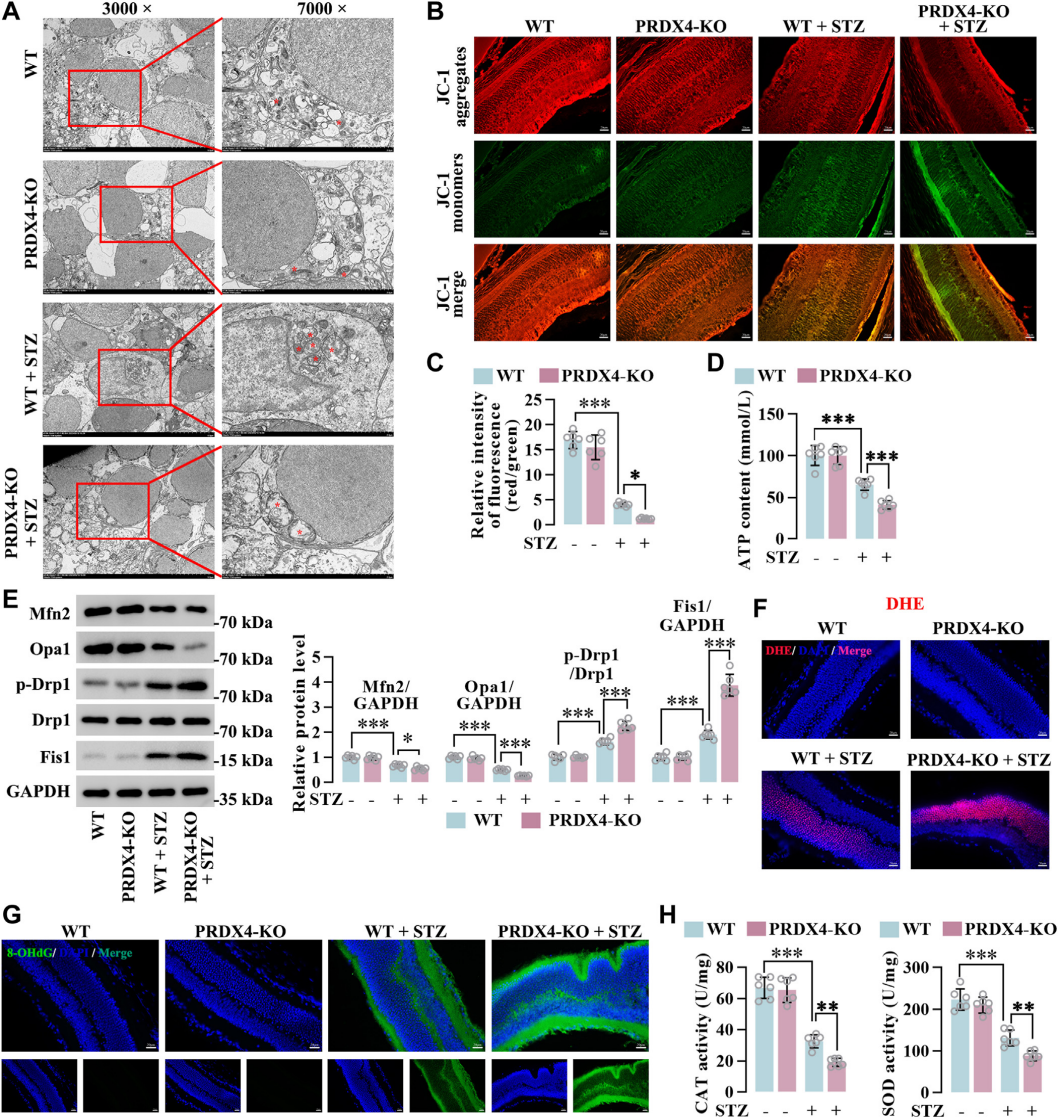
**PRDX4 deficiency contributed to mitochondria dysfunction and oxidative stress in the retinas of STZ-induced diabetic mice**. *A*, the morphology of mitochondria in retinas of diabetic WT and PRDX4-KO mice as observed by transmission electron microscopy. *Asterisks* indicate mitochondria. Scale bar represents 5 μm or 2 μm. *B* and *C*, mitochondrial membrane potential in the mitochondrial fraction from retinas of WT and PRDX4-KO mice under nondiabetic and diabetic conditions was measured using the JC-1. *D*, ATP content in retinas of nondiabetic and diabetic WT and PRDX4-KO mice. *E*, representative immunoblots of mitochondrial fusion proteins (Opa1 and Mfn2) and mitochondrial fission proteins (Fis1 and phosphorylated Drp1) in retinas. *F*, the DHE staining of retinas of nondiabetic and diabetic WT and PRDX4-KO mice. Scale bar represents 20 μm. *G*, immunofluorescence staining for 8-OHdG of retinas. Scale bar represents 20 μm. *H*, the levels of CAT and SOD in retinas of nondiabetic and diabetic WT and PRDX4-KO mice. All data are shown as mean ± SD. ∗*p* < 0.05, ∗∗∗*p* < 0.001 by one-way ANOVA with Tukey multiple comparison test. 8-OHdG, 8-hydroxy-2'-deoxyguanosine; CAT, catalase; DHE, dihydroethidium; PRDX4, peroxiredoxin 4; SOD, superoxide dismutase; STZ, streptozotocin.

To gain a broader understanding of the molecular pathology of PRDX4 deficiency–induced retinal injury in diabetes, the regulatory effects of PRDX4 deficiency on mitochondrial function were evaluated. In the WT mice and PRDX4-KO mice, the mitochondrial structure of retinal cells was normal. Retinal cells in diabetic mice displayed changes in mitochondrial morphology marked by swollen mitochondria with disrupted cristae. In retinas of diabetic PRDX4-KO mice, the reduced mitochondria were more swollen and had more broken or disappeared cristae (Fig. 3*A*). We found that STZ induction reduced red fluorescence intensity and enhanced green fluorescence intensity compared with the WT mice, indicating loss of mitochondrial membrane potential. PRDX4-KO aggravated the loss of mitochondrial membrane potential in retinas of diabetic mice, as evidenced by the lower ratio of red to green fluorescence (Fig. 3, *B* and *C*). The assessment of ATP content in retinas suggested that STZ induced a reduction in ATP production, whereas PRDX4-KO in diabetic mice decreased it even more (Fig. 3*D*). We then explored the impact of PRDX4 on mitochondrial dynamics of retinas under diabetic conditions. The expression of mitochondrial fusion–associated genes (Mfn2 and Opa1) was decreased by STZ treatment. Conversely, the levels of mitochondrial fission–associated marks (Fis1 and phosphorylated Drp1) were increased under high diabetic conditions. PRDX4 deficiency led to further decrease in levels of mitochondrial fusion–related genes and further enhancement in mitochondrial fission–related genes (Fig. 3*E*). These results elaborated that PRDX4 deficiency contributed to mitochondria dysfunction and mitochondrial fission in the retinas of diabetic mice.

Considering that oxidative stress results in mitochondrial dysfunction, we further investigated the effects of PRDX4 deficiency on oxidative stress in the retinas of diabetic mice. Superoxide production in the retina was detected using dihydroethidium (DHE) probe. The retinas of diabetic mice showed stronger oxidized DHE fluorescence than WT mice or PRDX4-KO mice. PRDX4-KO in diabetic mice resulted in more fluorescence in the retinas than diabetic mice (Fig. 3*F*). Immunofluorescent staining of 8-hydroxy-2'-deoxyguanosine (8-OHdG), an oxidative DNA damage marker, illustrated that no 8-OHdG signal was observed in the retinas of WT mice or PRDX4-KO mice. An increase of 8-OHdG signal was observed in the retinas of diabetic mice. This increase was further enhanced in the retinas of diabetic PRDX4-KO mice (Fig. 3*G*). Diabetic mice showed relatively lower levels of catalase (CAT) and superoxide dismutase (SOD) in the retinas compared with WT mice or PRDX4-KO mice. PRDX4-KO in diabetic mice exhibited further adverse impacts on the production of CAT and SOD (Fig. 3*H*). These results suggested that PRDX4 deficiency aggravated oxidative stress in the retinas of diabetic mice.

### PRDX4 inhibited reactive gliosis, apoptosis, mitochondria dysfunction, oxidative stress, and ER stress of HG-induced Müller cells

**Figure 4:**
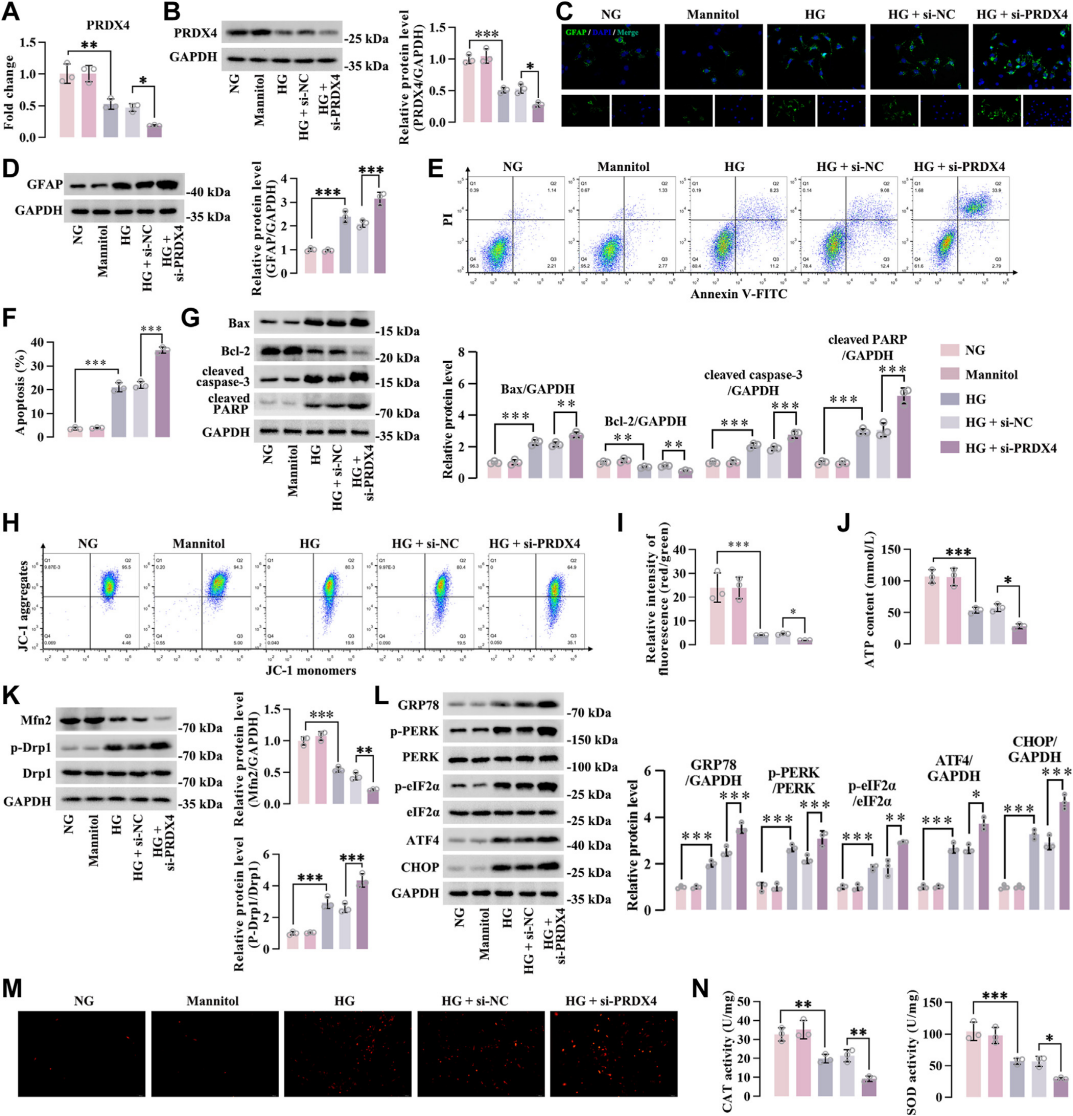
**Knockdown of PRDX4 promoted reactive gliosis, apoptosis, mitochondria dysfunction, oxidative stress, and ER stress of HG-induced Müller cells**. PRDX4-silenced Müller cells were incubated under HG conditions for 24 h. *A* and *B*, PCR and Western blot analysis of PRDX4 in Müller cells under different treatments. *C* and *D*, immunofluorescence staining and Western blot analysis of GFAP in Müller cells. Scale bar represents 50 μm. *E* and *F*, flow cytometry analysis of apoptotic Müller cells under different treatments. *G*, protein levels of Bax, cleaved caspase-3, cleaved PARP, and Bcl-2 in Müller cells after HG exposure. *H* and *I*, mitochondrial membrane potential in Müller cells. *J*, ATP content in Müller cells under HG conditions. *K*, the changes of mitochondrial fission and fusion proteins in Müller cells after HG exposure. *L*, expression levels of ER stress–associated proteins in Müller cells. *M*, representative images of Müller cells after different treatments with DHE staining. Scale bar represents 100 μm. *N*, the activities of CAT and SOD in Müller cells following different treatments. ∗*p* < 0.05, ∗∗*p* < 0.01, ∗∗∗*p* < 0.001 by one-way ANOVA with Tukey multiple comparison test. CAT, catalase; DHE, dihydroethidium; ER, endoplasmic reticulum; GFAP, glial fibrillary acidic protein; HG, high glucose; PARP, poly(ADP-ribose) polymerase; PRDX4, peroxiredoxin 4; SOD, superoxide dismutase.

To confirm the regulation of PRDX4 on the aforementioned events *in vitro*, a knockdown system was developed in Müller cells in which the expression of endogenous PRDX4 was silenced by siRNAs. We selected Müller cells transfected with siRNA-mediated PRDX4-3 (si-PRDX4-3) for HG stimulation, as the knockdown effect of this siRNA was most significant (Fig. S1*A*). There was a decrease in PRDX4 expression both at mRNA and protein levels under HG conditions, and knockdown of PRDX4 led to a further reduction of PRDX4 expression in HG-induced Müller cells (Fig. 4, *A* and *B*). Western blot and immunofluorescence analysis indicated that the expression of GFAP was increased in HG-induced Müller cells, and its expression was further enhanced in PRDX4-silenced Müller cells upon HG treatment (Fig. 4, *C* and *D*). PRDX4 silence resulted in increased apoptotic rate of Müller cells under HG conditions (Fig. 4, *E* and *F*). The proapoptotic effects of PRDX4 siRNA in HG-induced Müller cells was verified, as evidenced by increased Bax, cleaved caspase-3, and cleaved PARP level, and decreased Bcl-2 expression (Fig. 4*G*). Compared with HG-induced Müller cells transfected with siRNA negative control (si-NC), mitochondrial membrane potential and ATP content were reduced in PRDX4-silenced Müller cells under HG conditions (Fig. 4, *H*–*J*). We also found reduction of Mfn2 expression and enhancement of Drp1 phosphorylation level in PRDX4-silenced Müller cells compared with Müller cells transfected with si-NC under HG conditions (Fig. 4*K*). The levels of ER stress–related markers (GRP78, ATF4, CHOP, as well as the phosphorylated PERK and eIF2α) were higher in Müller cells transfected with PRDX4 siRNA than those transfected with si-NC after HG treatment (Fig. 4*L*). After HG stimulation, Müller cells had a substantial increase in DHE staining intensity, which was further enhanced in PRDX4-silenced Müller cells under HG conditions (Fig. 4*M*). HG treatment repressed the activities of CAT and SOD in Müller cells, and this inhibitory effect was amplified by PRDX4 siRNA (Fig. 4*N*). In contrast, ectopic expression of PRDX4 in Müller cells inhibited reactive gliosis, apoptosis, mitochondria dysfunction, ER stress, and oxidative stress under HG conditions (Figs. S1*B*, and 5). Collectively, these data indicated that PRDX4 inhibited reactive gliosis, apoptosis, mitochondria dysfunction, ER stress, and oxidative stress of HG-induced Müller cells.

**Figure 5:**
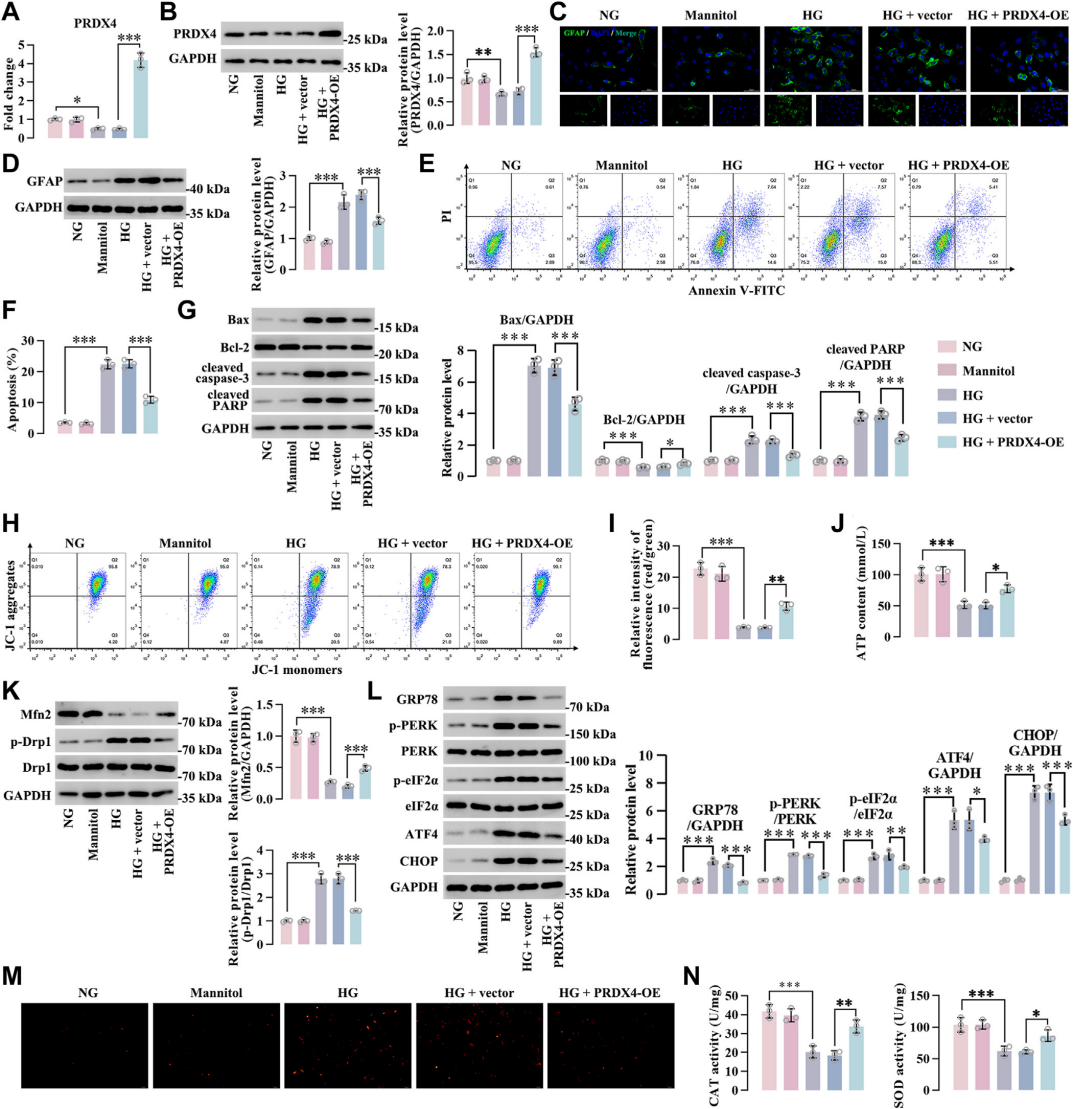
**PRDX4 overexpression inhibited reactive gliosis, apoptosis, mitochondria dysfunction, oxidative stress, and ER stress of HG-induced Müller cells**. PRDX4-overexpressed Müller cells were incubated under HG conditions for 24 h. *A* and *B*, PCR and Western blot analysis of PRDX4 in Müller cells under different treatments. *C* and *D*, immunofluorescence staining and Western blot analysis of GFAP in Müller cells. Scale bar represents 50 μm. *E* and *F*, flow cytometry analysis of apoptotic Müller cells under different treatments. *G*, protein levels of Bax, cleaved caspase-3, cleaved PARP, and Bcl-2 in Müller cells after HG exposure. *H* and *I*, mitochondrial membrane potential in Müller cells. *J*, ATP content in Müller cells under HG conditions. *K*, the changes of mitochondrial fission and fusion proteins in Müller cells after HG exposure. *L*, expression levels of ER stress–associated proteins in Müller cells. *M*, representative images of Müller cells after different treatments with DHE staining. Scale bar represents 100 μm. *N*, the activities of CAT and SOD in Müller cells following different treatments. ∗*p* < 0.05, ∗∗*p* < 0.01, ∗∗∗*p* < 0.001 by one-way ANOVA with Tukey multiple comparison test. CAT, catalase; DHE, dihydroethidium; ER, endoplasmic retciulum; GFAP, glial fibrillary acidic protein; HG, high glucose; PARP, poly(ADP-ribose) polymerase; PRDX4, peroxiredoxin 4; SOD, superoxide dismutase.

### PRDX4 alleviated HG-stimulated Müller cell abnormalities by destabilizing DPP4 protein stability

**Figure 6:**
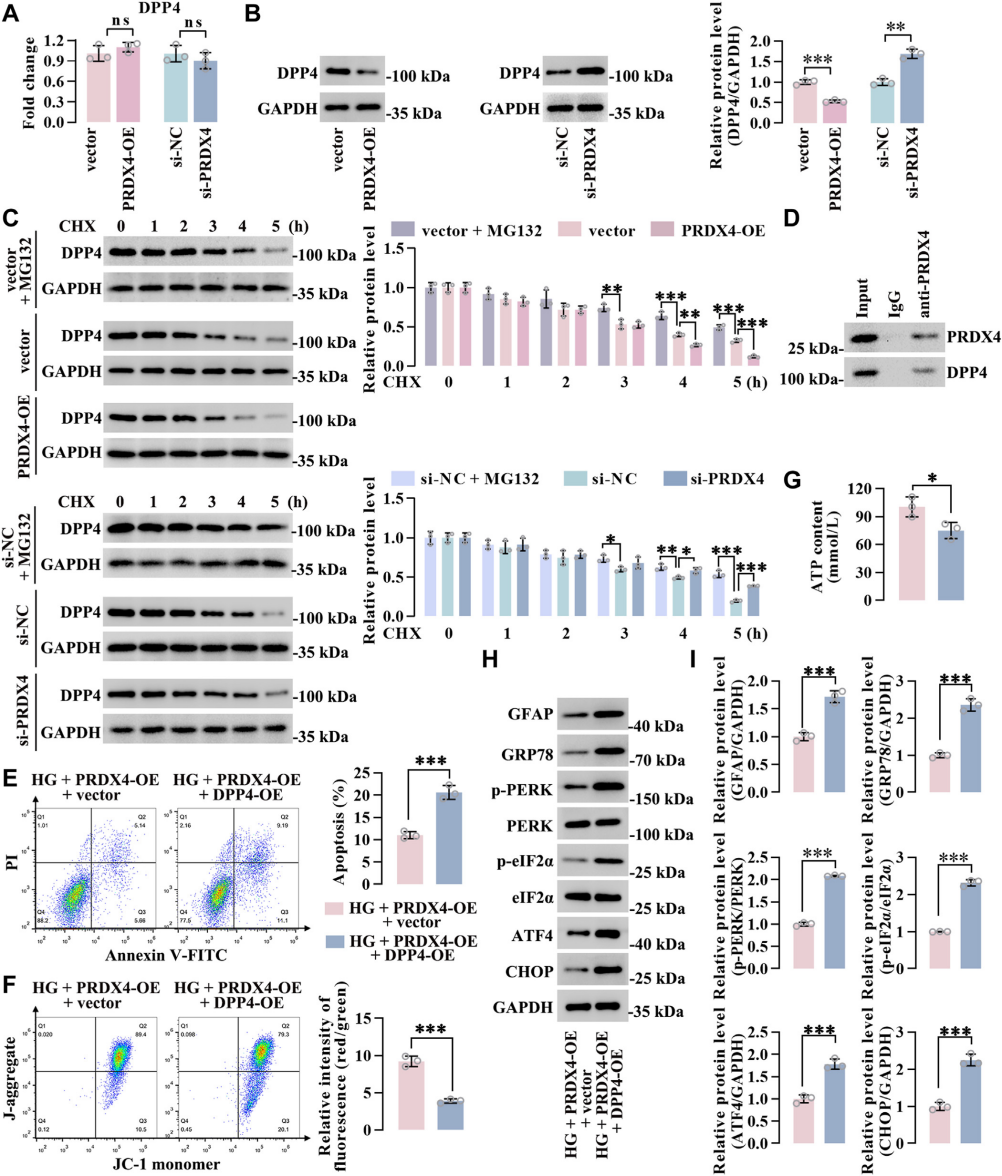
**PRDX4 alleviated HG-stimulated Müller cell abnormalities by destabilizing DPP4 protein stability**. *A* and *B*, the mRNA and protein levels of DPP4 in PRDX4 overexpressing or silencing Müller cells. *C*, protein stability assay for DPP4 in PRDX4 overexpressing or silencing Müller cells treated with CHX (100 μg/ml) for the indicated time points. *D*, coimmunoprecipitation assay showed the interaction between PRDX4 and DPP4 in Müller cells. *E*–*I*, PRDX4 overexpression plasmid and DPP4 overexpression plasmid were cotransfected into Müller cells under HG conditions. Flow cytometry was performed to investigate the apoptosis rate of Müller cells (*E*). *F* and *G*, mitochondrial membrane potential and ATP content in Müller cells. *H* and *I*, expression levels of GFAP and ER stress–associated proteins in Müller cells. ∗*p* < 0.05, ∗∗*p* < 0.01, ∗∗∗*p* < 0.001 by Student’s *t* test and one-way ANOVA with Tukey multiple comparison test. CHX, cycloheximide; DPP4, dipeptidyl peptidase-4; ER, endoplasmic reticulum; GFAP, glial fibrillary acidic protein; HG, high glucose; PRDX4, peroxiredoxin 4.

Subsequently, we investigate the molecular mechanism by which PRDX4 confers protective effects against DR. Considering that PRDX4 participates to regulate the protein stability of β-catenin (33), the mechanism underlying the beneficial effects of PRDX4 may be linked to modulation of protein stability in Müller cells. From the BioGrid protein interaction database (https://thebiogrid.org/), we found that PRDX4 interacts with several proteins, including DPP4, which is associated with DR in diabetics, and its inhibition is beneficial for DR (30, 31, 32). PRDX4 overexpression resulted in a decrease in DPP4 protein level, and PRDX4 knockdown led to an increase in DPP4 protein expression, and PRDX4 overexpression or silencing did not alter DPP4 transcript level in Müller cells (Fig. 6, *A* and *B*), implying the post-transcriptional regulation of DPP4 by PRDX4. To verify the potential that PRDX4 promotes the instability of DPP4 protein, PRDX4-overexpressed or PRDX4-silenced Müller cells were subject to cycloheximide (100 μg/ml) treatment for protein synthesis inhibition, followed by monitoring of DPP4 protein levels. As a positive control, Müller cells transfected with vector plasmid or si-NC were incubated with 5 μM MG132 (the proteasome inhibitor) for 8 h, then treated with cycloheximide. MG132 treatment enhanced the stability of DPP4 at all time points. After PRDX4 overexpression, DPP4 protein was highly unstable, and DPP4 protein degradation was reduced upon PRDX4 knockdown (Fig. 6*C*). Moreover, coimmunoprecipitation assay indicated a direct interaction between PRDX4 and DPP4 in Müller cells (Fig. 6*D*).

The role of DPP4 in HG-induced Müller cells was explored by the knockdown system using si-DPP4. Knockdown of DPP4 inhibited reactive gliosis, apoptosis, mitochondria dysfunction, ER stress, and oxidative stress in HG-induced Müller cells (Fig. S2). We then investigated whether PRDX4 influences cellular events of HG-induced Müller cells by downregulating DPP4. DPP4 overexpression plasmid was transfected into Müller cells, and the transfection effect was detected by Western blot analysis (Fig. S1*C*). When PRDX4 overexpression plasmid and DPP4 overexpression plasmid were cotransfected into Müller cells, the phenotypes following PRDX4 overexpression alone were reversed by DPP4 overexpression, including increased apoptosis, mitochondria dysfunction (as evidenced by diminished mitochondrial membrane potential and ATP content), reactive gliosis (as evidenced by increased GFAP expression), and ER stress (as evidenced by enhanced levels of GRP78, phosphorylated-PERK, phosphorylated-eIF2α, ATF4, and CHOP) under HG conditions (Fig. 6, *E*–*I*). We concluded that PRDX4 alleviated HG-stimulated Müller cell abnormalities by destabilizing DPP4 protein stability.

## Discussion

In this study, we have shown for the first time that PRDX4 deficiency accelerated pathological changes of retina in STZ-induced diabetic mice through augmenting reactive gliosis of Müller cells, cell apoptosis, ER stress, mitochondrial dysfunction, and oxidative stress. Our studies in HG-induced Müller cells consolidated the protective role of PRDX4 against the aforementioned cellular events. Mechanistically, PRDX4 destabilized protein stability of DPP4, thereby alleviating HG-stimulated Müller cell abnormalities. However, the specific mechanism by which PRDX4 regulates DPP4 stability is not currently well understood. Wang *et al*. (33) reported that PRDX4 augments β-catenin protein stability by interacting with ubiquitin ligase β-TrCP and preventing β-TrCP-mediated β-catenin ubiquitination. Based on their findings, we hypothesized that an interaction between PRDX4 and a deubiquitinase may hinder the deubiquitinase's ability to bind to DPP4, leading to the destabilization of DPP4 protein. This possibility requires further investigation.

Retinal neurodegeneration encompasses features of glial cell activation, an early event in the pathogenesis of DR (34). Moreover, reactive gliosis hinders regenerative process in retina through forming a glial scar (35). Müller cells, which serve as the principal glial cells in the retina, play a crucial role in maintaining retinal homeostasis. Hyperglycemia-caused retinal damage drives Müller cells to become active, characterized by the upregulation of GFAP expression (36). Reactive Müller cells secrete deleterious cytokines that modulate the inflammatory response to facilitate the development of DR (37). The blockage of reactive gliosis could be a promising therapeutic strategy for alleviating DR lesions. Our findings demonstrated that GFAP expression in Müller cells increases as diabetes progresses and provided the first evidence that PRDX4 suppressed reactive gliosis of Müller cells during DR.

ER is a subcellular organelle that ensures proper protein folding, lipid biosynthesis, and maintenance of calcium homeostasis (38). Disturbance in ER function leads to ER stress and subsequent activation of the unfolded protein response. Emerging evidence identifies ER stress as a key sensor in the regulation of inflammation, cellular energy metabolism, redox state, and cell survival in diabetes (39, 40, 41). ER stress induced by hyperglycemia in Müller cells has been linked to DR progression (42). Under ER stress, accumulation of unfolded proteins in the ER leads to the dissociation of the ER chaperone GRP78 from ER stress sensors, initiating the activation of unfolded protein response branches (43). One of these branches is the PERK pathway, which is triggered by PERK phosphorylation. Activated PERK phosphorylates eIF2α and promotes ATF4 translation. In turn, ATF4 induces the transcription of CHOP, a proapoptotic transcription factor. Although the levels of proteins involved in PERK pathway were increased in retina of diabetic mice, with higher levels observed in STZ-treated PRDX4-KO mice, indicating PRDX4 alleviated ER stress in retina of diabetic mice. Consistent with this finding, *in vitro* results demonstrated that PRDX4 mitigated HG-induced ER stress in Müller cells. Thus, we concluded that PRDX4 reduced Müller cell apoptosis partially by inhibiting ER stress–related pathways, thereby conferring protection from retinopathic changes. Study conducted by Elko *et al*. (44) proposed a link between PRDX4 and ER stress. Specifically, HSPA5, an initiator of the ER stress response, binds PRDX4 under oxidizing conditions, indicating a possible putative role of PRDX4 oxidation in triggering ER stress. The potential mechanism of PRDX4 in regulating ER stress in Müller cells under HG stimulation warrants further investigation.

The retina consumes substantial amounts of glucose and oxygen to produce ATP for its phototransduction and visual functions (45). Thus, preserving the integrity and homeostasis of mitochondria is essential, accomplished by a balance between mitochondrial fusion and fission. Mitochondrial fusion merges two mitochondria at the outer and inner membrane interfaces *via* three membrane GTPases (Mfn1, Mfn2, and Opa1) to derive a healthy and distinct mitochondrion. Mitochondrial fission is coordinated by a GTPase, Drp1, which is recruited to the mitochondria by its adaptors (including MFF, MID49, MID51, and Fis1) present on the outer mitochondrial membrane (46). Mitochondrial dynamics is associated with some cellular events, including apoptosis (47). In this study, we explored the impact of genetic manipulation of PRDX4 on mitochondrial function and its consequent influence on Müller cell apoptosis. Our findings indicated that mitochondrial fission appeared to be dominant during HG stimulation. Fragmented mitochondria were inefficient in ATP production, leading to decreased mitochondrial membrane potential. These changes are concomitant with apoptosis of Müller cells, indicating a potential involvement of mitochondrial dysfunction in HG-induced Müller cell apoptosis. The improvement of mitochondrial function with PRDX4 caused an inhibition of apoptosis of HG-treated Müller cells; these regulatory effects were corroborated in the retinas of diabetic PRDX4-KO mice.

In conclusion, our study suggested that PRDX4 reduces Müller cell apoptosis, alleviates reactive gliosis, maintains ER homeostasis, restores mitochondrial function, and suppresses oxidative stress of Müller cells under hyperglycemia, which is a potential protective mechanism in DR. Our work contributes novel insights to the field of translational medicine in the context of DR intervention.

## Experimental procedures

### Animals

Male C57BL/6 WT mice were procured from Liaoning Changsheng Biotechnology Co, Ltd. PRDX4-KO mice were generated by GemPharmatech Co, Ltd using the CRISPR-associated Cas9 nuclease (CRISPR/Cas9) genome editing technique.

The diabetic model was established in mice by intraperitoneal injection of STZ (Aladdin regents Co Ltd; dissolved in 0.1 mol/l of citrate buffer) at a dose of 50 mg/kg for 5 consecutive days. The control mice were injected with an equivalent volume of citrate buffer. Mice were deemed diabetic once their blood glucose levels >16.7 mmol/l. After the last injection of STZ, changes in body weight and blood glucose were monitored biweekly. At week 12 after successful modeling of diabetes, mice were euthanized and retinal tissues were collected for subsequent experiments. The animal study was approved by the Ethics Committee of Shengjing Hospital of China Medical University and performed in compliance with applicable guidelines and regulations.

### Cell culture and treatment

Human Müller cells (Procell Life Science&Technology Co, Ltd) were cultured in Dulbecco's modified Eagle's medium (ThermoFisher Scientific) containing 10% fetal bovine serum and 1% penicillin–streptomycin in a chamber with 5% CO_2_ at 37°C. Müller cells were subjected to various conditions: 25 mM glucose (HG), 5.5 mM glucose + 19.5 mM mannitol (as an osmotic control), and 5.5 mM glucose (normal glucose).

### Cell transfection

pcDNA3.1-3xFlag-PRDX4 was constructed by inserting an open reading frame of PRDX4 into pcDNA3.1-3xFlag vector to overexpress PRDX4 (YouBio Biology). pcDNA3.1-3xFlag-DPP4 was constructed by inserting an open reading frame of DPP4 into pcDNA3.1-3xFlag vector to overexpress DPP4 (YouBio Biology). si-PRDX4 si-NC were obtained from GenePharma. Lipofectamine 3000 (Invitrogen) was employed to transfect PRDX4–DPP4 overexpression plasmid or si-PRDX4 into Müller cells. Cells were exposed to different treatment 24 h post-transfection.

### Western blot analysis

Retinal tissues or Müller cells were lysed with RIPA Lysis Buffer (Beyotime Biotechnology). Protein concentration was measured by BCA Protein Assay Kit (Beyotime Biotechnology). Proteins loaded at 30 μg per lane were resolved *via* SDS-PAGE and then transferred onto polyvinylidene fluoride membranes. Following blocking with 5% nonfat milk, the membranes were subjected to incubation with the following primary antibodies: anti-PRDX4 (10703-1-AP; Proteintech Group), anti-GS (ab228590; Abcam), anti-GFAP (ab279291; Abcam), anti-Bax (50599-2-Ig; Proteintech Group), anti-Bcl-2 (26593-1-AP; Proteintech Group), anti–cleaved caspase-3 (#9661; Cell Signaling Technology), anti-cleaved PARP (#9541; Cell Signaling Technology), anti-GRP78 (ab21685; Abcam), anti-CHOP (15204-1-AP; Proteintech Group), anti-phospho-PERK (Thr982) (82534-1-RR; Proteintech Group), anti-PERK (24390-1-AP; Proteintech Group), anti-phospho-eIF2α (Ser51) (68023-1-Ig; Proteintech Group), anti-eIF2α (11170-1-AP; Proteintech Group), anti-ATF4 (10835-1-AP; Proteintech Group), anti-Mfn2 (ab124773; Abcam), anti-phospho-Drp1 (Ser616) (ab314755; Abcam), anti-Drp1 (ab184247; Abcam), anti-Opa1 (ab157457; Abcam), anti-Fis1 (ab229969; Abcam), anti-DPP4 (MA5-32643; ThermoFisher Scientific), and anti-GAPDH (#2118; Cell Signaling Technology) overnight at 4°C. The membranes were probed with antirabbit or antimouse IgG (#7074, #7076; Cell Signaling Technology) for 1.5 h at room temperature. Subsequently, the detection of immunoreactive bands was performed using the ECL kit (Beyotime Biotechnology). The blots were scanned and analyzed by Image J software (National Institutes of Health). The intensity of each band was normalized to GAPDH.

### H&E staining

Sections of retinal tissues were staining with H&E to observe retinal morphology at magnifications of 200× and 400×. The thickness of retina, outer nuclear layer, and inner nuclear layer, coupled with the number of cells in ganglion cell layer, was quantified.

### Immunofluorescence staining

For paraffin-embedded tissues, sections of 5 μm were prepared, deparaffinized in xylene, rehydrated in graded alcohol, and subjected to antigen retrieval using citric acid buffer. Müller cells were fixed using 4% paraformaldehyde solution and permeabilized with 0.1% Triton X-100. Afterward, retina or cell sections were subjected to an overnight incubation at 4°C with primary antibodies against GFAP (ab279291; Abcam) and GS (ab228590; Abcam) or primary antibody against 8-OHdG (sc-66036; Santa Cruz Biotechnology). Following PBS washing, the sections were exposed to Goat Anti-Rat IgG and/or Goat Anti-Rabbit IgG (Abcam) for 2 h. 4′,6-diamidino-2-phenylindole was employed for labeling cell nuclei. The sections were examined with a microscope (Olympus) at a magnification of 400×.

### TUNEL staining

TUNEL staining was conducted to detect apoptotic cells within retinal tissues. A Tunel Cell Apoptosis Detection Kit (G1504; Servicebio) was utilized following the manufacturer's instruction. Following staining with 4′,6-diamidino-2-phenylindole, the slides were examined with a microscope (Olympus).

### Transmission electron microscopy

To observe mitochondria morphology, retinal tissues were fixed in 2.5% glutaraldehyde (Beijing Solarbio Science & Technology Co, Ltd) and 1% osmium tetroxide (Beijing Zhongjingkeyi Technology Co, Ltd). After gradient dehydration, specimens underwent embedding and sectioning accompanied by staining using uranyl acetate (Beijing Zhongjingkeyi Technology Co, Ltd) and lead citrate (Beijing Zhongjingkeyi Technology Co, Ltd). The samples were photographed using a transmission electron microscope (Hitachi).

### Determination of mitochondrial membrane potential

Mitochondrial membrane potential in retinas or Müller cells was measured by JC-1 Mitochondrial Membrane Potential Kit (C2006; Beyotime Biotechnology). Fluorescence intensity of JC-1 monomers/aggregates (green fluorescence for monomers and red fluorescence for aggregates) was examined by a fluorescence microscopy (Olympus) or a flow cytometer (BD).

### Determination of ATP content

ATP content was detected by the ATP assay kit (S0026; Beyotime Biotechnology). Retinas were homogenized in lysis buffer. Müller cells were lysed with lysis buffer. After centrifugation, the supernatants were retrieved and mixed with ATP assay working solution. Luminescence was measured by a microplate luminometer (Safire II, Tecan). Standard curves were prepared, and protein concentrations in samples were determined with the BCA Protein Concentration Assay Kit (P0009; Beyotime Biotechnology).

### Detection of reactive oxygen species production

DHE (MedChemExpress) staining for reactive oxygen species was performed on retina sections or Müller cells. Frozen sections of retina tissues were incubated with 7.5 mM DHE for 30 min at 37°C in the dark. Müller cells were incubated with 5 μM DHE in serum-free medium for 30 min. After being rinsed in PBS, fluorescence was visualized under a microscopy (Olympus).

### Measurement of antioxidative enzymes

According to the manufacturer’s instructions, the activities of CAT and SOD in retina tissues or Müller cells were detected with the corresponding commercial kits purchased from Nanjing Jiancheng Bioengineering Institute or Beijing Solarbio Science & Technology Co, Ltd.

### Flow cytometry assay

Müller cells were collected using EDTA free trypsin and resuspended in PBS. After centrifugation, cells were then stained with 5 μl Annexin V-FITC and 5 μl propidium iodide (Tianjin Sungene Biotech Co, Ltd). Apoptosis was detected using a flow cytometer (BD).

### Reverse transcription quantitative PCR

RNA was isolated with the TriQuick Reagent (Beijing Solarbio Science & Technology Co, Ltd) and reversely transcribed using the RT-PCR Kit (Beijing Solarbio Science & Technology Co, Ltd). PCR amplification was performed with SYBR Green I (Beijing Solarbio Science & Technology Co, Ltd) in the Real-Time PCR System (ThermoFisher Scientific). Primers employed are listed as follows: PRDX4, Forward 5′-GCAAAGCGAAGATTTCCAAG-3′, Reverse 5′-GGCCAAATGGGTAAACTGTG-3'; DPP4, Forward 5′-ATGCCAGGAGGAAGGAATCT-3′, Reverse 5′-TATAGAGGGGCAGACCAGGA-3'. The relative mRNA level was quantified using 2^−ΔΔCt^ method.

### Coimmunoprecipitation assay

Cellular lysates were incubated with 5 μg of anti-PRDX4 (10703-1-AP; Proteintech Group) or anti-IgG (#3900; Cell Signaling Technology) antibodies overnight at 4°C. The mixture was captured with 35  μl of protein A/G agarose beads (Beijing Solarbio Science & Technology Co, Ltd) for 2 h at room temperature. Afterward, the immunoprecipitates were eluted from the beads and subjected to Western blot analysis.

### Statistical analysis

GraphPad Prism 9.0 software was employed to conduct statistical analysis. The results were represented as the mean ± SD. Unpaired *t* test was employed to compare two groups, one-way ANOVA was utilized for comparisons among multiple groups, and two-way ANOVA was utilized for comparisons of body weight or blood glucose. Statistical significance was inferred when the *p* value <0.05.

## Data availability

The BioGrid protein interaction database used in the current study is available in https://thebiogrid.org/.

## Supporting information

This article contains supporting information.

## Conflict of interest

The authors declare that they have no conflicts of interest with the contents of this article.
