# Supplementary material for: PRDX4 mitigates diabetic retinopathy by inhibiting reactive gliosis, apoptosis, ER stress, oxidative stress, and mitochondrial dysfunction in Müller cells
Source: J Biol Chem. 2024 Dec 18;301(1):108111. doi: 10.1016/j.jbc.2024.108111 (PMC11760821; doi:10.1016/j.jbc.2024.108111)
Supplement: Supplemental Figs. S1 and S2 [file mmc1.docx]

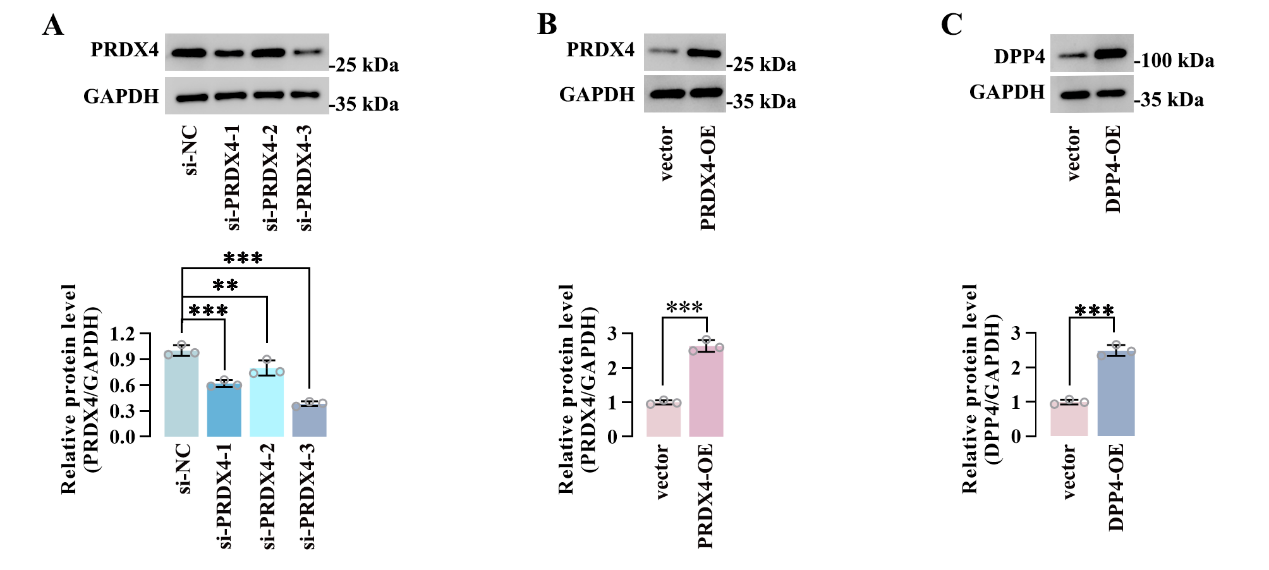


**Supplementary Figure 1**

(A, B) Immunoblots of PRDX4 in PRDX4 siRNAs or PRDX4 overexpression plasmid transfected Müller cells. (C) Immunoblots of DPP4 in DPP4 overexpression plasmid transfected Müller cells.


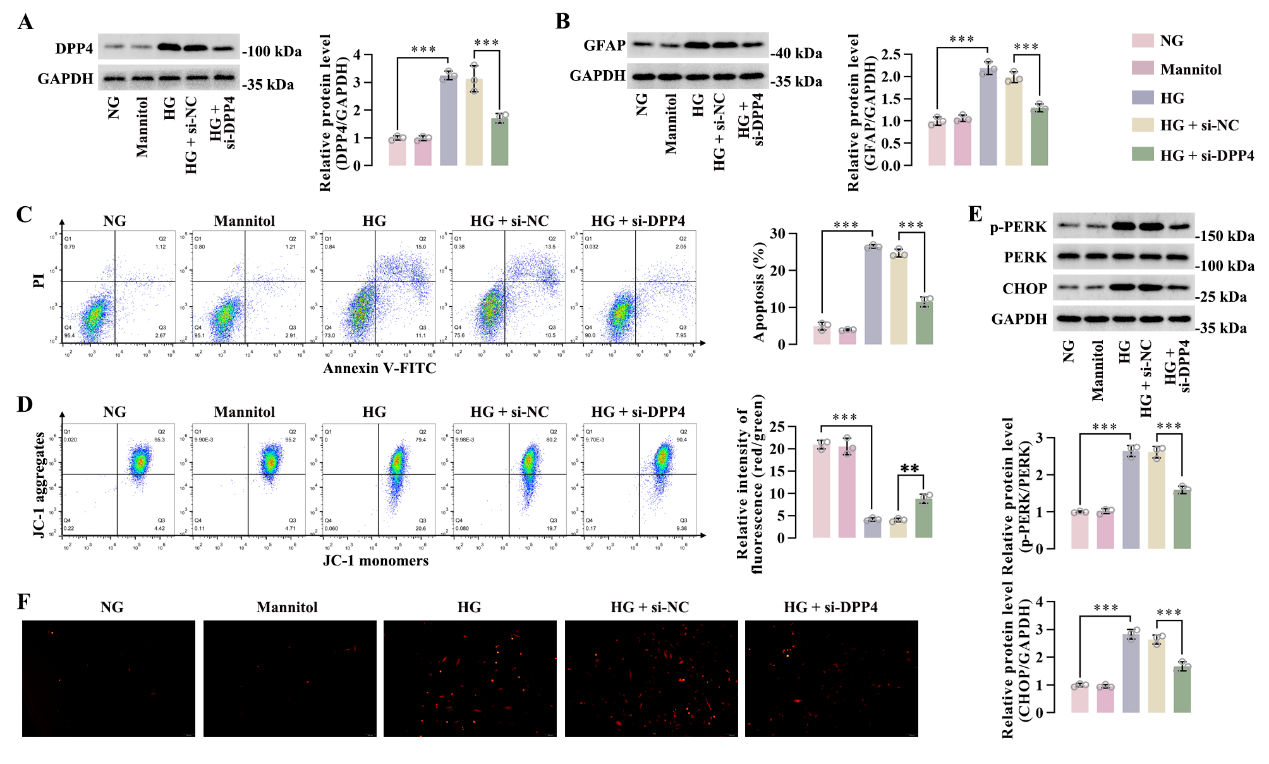


**Supplementary Figure 2**

DPP4-silenced Müller cells were incubated under HG conditions for 24 h. (A) Western blot analysis of DPP4 in Müller cells under different treatments. (B) Western blot analysis of GFAP in Müller cells. (C) Flow cytometry analysis of apoptotic Müller cells under different treatments. (D) Mitochondrial membrane potential in Müller cells under HG conditions. (E) Expression levels of ER stress-associated proteins in Müller cells. (F) Representative images of Müller cells after different treatments with DHE staining. Scale bar = 100 μm. *P < 0.05, ** P < 0.01, ***P < 0.001 by one-way ANOVA with Tukey’s multiple comparison test.
